# Cerebrospinal Fluid Biomarker Profile in TDP-43-Related Genetic Frontotemporal Dementia

**DOI:** 10.3390/jpm12101747

**Published:** 2022-10-21

**Authors:** Elisabeth Kapaki, Foteini Boufidou, Mara Bourbouli, Efstratios-Stylianos Pyrgelis, Vasilios C. Constantinides, Cleo Anastassopoulou, George P. Paraskevas

**Affiliations:** 1 1st Department of Neurology, School of Medicine, National and Kapodistrian University of Athens, Eginition Hospital, 74, Vass. Sophias Ave., 11528 Athens, Greece; 2 Neurochemistry and Biological Marker Unit, 1st Department of Neurology, School of Medicine, National and Kapodistrian University of Athens, Eginition Hospital, 74, Vass. Sophias Ave., 11528 Athens, Greece; 3 Department of Microbiology, Medical School, National and Kapodistrian University of Athens, 75 Mikras Asias Street, 11527 Athens, Greece; 4 2nd Department of Neurology, School of Medicine, National and Kapodistrian University of Athens, Attikon University Hospital, Rimini 1, 12462 Athens, Greece

**Keywords:** frontotemporal dementia, genetic FTD, TDP-43 proteinopathy, cerebrospinal fluid, biomarkers, *C9orf72*, *GRN*, *VCP*

## Abstract

Cerebrospinal fluid (CSF) biomarkers, namely total tau, phospho-tau and amyloid beta peptides, have received much attention specifically regarding Alzheimer’s disease (AD), since they can detect the biochemical fingerprint of AD and serve as a diagnostic tool for accurate and early diagnosis during life. In the same way, biomarkers for other neurodegenerative disease pathologies are also needed. We present a case series of six patients with genetic frontotemporal dementia (FTD), with TDP-43 underlying proteinopathy, in an attempt to assess TDP-43 as a novel biomarker alone and in combination with established AD biomarkers for this specific patient group, based on the principles of personalized and precision medicine. Our results indicate that genetic TDP-43-FTD is characterized by increased CSF TPD-43 and increased TDP-43 × τ_Τ_/τ_P-181_ combination. Hence, TDP-43 combined with tau proteins could be a useful tool for the diagnosis of genetic FTD with TDP-43 underling histopathology, supplementing clinical, neuropsychological and imaging data.

## 1. Introduction

A common neuropathological feature of neurodegenerative diseases is cytoplasmic protein aggregates. Cytoplasmic mislocalization and aggregation of TAR-DNA binding protein 43 (TDP-43) is found in the vast majority of patients with amyotrophic lateral sclerosis (ALS), and in approximately half of patients with frontotemporal dementia (FTD) with ubiquitin inclusions [1].

Recently, great progress in the field of neurogenetics has resulted in the recognition of several gene mutations related to the FTD-ALS spectrum, especially with ubiquitin-TDP-43 predominant histopathology, thus highlighting a new brain proteinopathy. The most recent, but more common discovery, is a genetic mutation in the *C9orf72* gene involving a hexanucleotide repeat expansion (GGGGCC) in the first non-coding intron of the gene, responsible for both familial and sporadic FTD and ALS, but more frequently for the combined FTD-ALS phenotype [2,3,4,5].

Heterozygous loss of function mutations in the progranulin gene (*GRN*) can also lead to FTD with TDP-43 inclusions [6]. GRN mutations have been linked to FTD phenotypes, particularly those of the primary progressive aphasia (PPA) subtype. Mutations in the valosin-containing protein (*VCP*) gene constitute another rare cause of FTD, which has been recognized to present with the clinical syndrome of “IBMPFD” (inclusion body myopathy, Paget’s disease of bone and FTD), often in combination with ALS in certain families. [7]. Mutations in the gene encoding the TDP-43 protein (*TARDBP*) are also an extremely rare cause of the FTD phenotype [8]. 

In recent years, cerebrospinal fluid (CSF) has proven to be an excellent material for biomarker discovery for neurodegenerative diseases [9]. Due to its close proximity to the brain parenchyma, CSF reflects the neurochemical changes taking place in the brain. This approach has been successful in the case of Alzheimer’s disease (AD), where tau proteins, total (τ_T_) and phosphorylated (τ_P_), as well as amyloid-β peptides (Aβ) expressing the pathological features of the disease, have been recognized as biomarkers with high diagnostic accuracy and are now included in research diagnostic criteria for AD [10,11,12,13,14,15]. The combination of increased τ_T_, tau phosphorylated at the threonine 181 site (τ_P-181_) and decreased Aβ with 42 amino acids (Aβ_42_) in the CSF, has currently been established as the biochemical signature of the disease in vivo [16]. 

Accordingly, the detection of TDP-43 could be a specific biomarker for TDP-43 proteinopathy. Attempts to quantify TDP-43 in CSF have been successful [17,18,19,20]. However, until now, TDP-43 seemed to lack adequate sensitivity and specificity in order to serve as a meaningful biomarker alone [21]. The combination of TDP-43 with the aforementioned AD biomarkers in the formula TDP-43 × τ_Τ_/τ_P-181_ may increase sensitivity and specificity (>80%) [18], while the τ_P-181_/τ_T_ ratio has been proposed as an indirect indicator of a non-tauopathic pathology [22,23].

Since almost all the aforementioned studies lack pathologic confirmation, we isolated and present herein a case series of six patients with genetic FTD and a presumed TDP-43 underlying proteinopathy. We aimed to investigate how the above-mentioned biomarkers perform in a personalized setting, based on the principles of a precision medicine clinical approach. We also include the clinical and imaging data of this group of patients.

## 2. Materials and Methods

A total of six patients were included in this case series study. All patients were hospitalized at the 1st Department of Neurology of the National and Kapodistrian University of Athens at Eginition Hospital during the years 2011–2018. The study was performed in accordance with the ethical guidelines of the Declaration of Helsinki, and had the approval of the local Ethical and Deontology committee of our hospital (244/5-7-2010). All study subjects and/or their relatives gave informed consent for inclusion in the study. 

Patients underwent detailed clinical, neuropsychological, biochemical and neuroimaging examination, to exclude secondary causes of dementia and establish an FTD diagnosis, based on the criteria of either Rascovsky et al. [24] for probable bvFTD or Gorno-Tempini et al. [25] for PPA. The Mini-Mental State examination (MMSE) and the Frontal Assessment Battery (FAB) were used as crude estimates of dementia severity [26,27].

The CSF levels of τ_T_, τ_P-181_ and Aβ_42_ and Aβ_40_ were measured in duplicate by commercially available enzyme-linked immunosorbent assay (ELISA) kits (“Innotest hTau antigen”, “phospho-tau181”, “β-amyloid1–42” and “β-amyloid1–40”, respectively, Fujirebio, Gent, Belgium) according to manufacturer’s instructions, and blind to clinical diagnosis. The above biomarkers are measured routinely in the Neurochemistry and Biomarker Unit of our department, as part of an everyday diagnostic approach, as described elsewhere [23]. TDP-43 was measured in triplicate by double sandwich ELISA, using a commercially available kit (Human TAR DNA-binding protein 43 ELISA kit, Cusabio Biotech Co., Ltd., Wuhan, China) according to manufacturer’s instructions, as described elsewhere [18]. The diagnostic performance of TDP-43 was assessed as a biomarker with molecular specificity in patients with an individualized FTD diagnosis, alone and combined with established AD biomarkers (mainly for excluding AD-related dementia). Cut-offs for normal values are shown in Table 1.

## 3. Results

### 3.1. Patients

#### 3.1.1. Case 1

**Figure 1:**
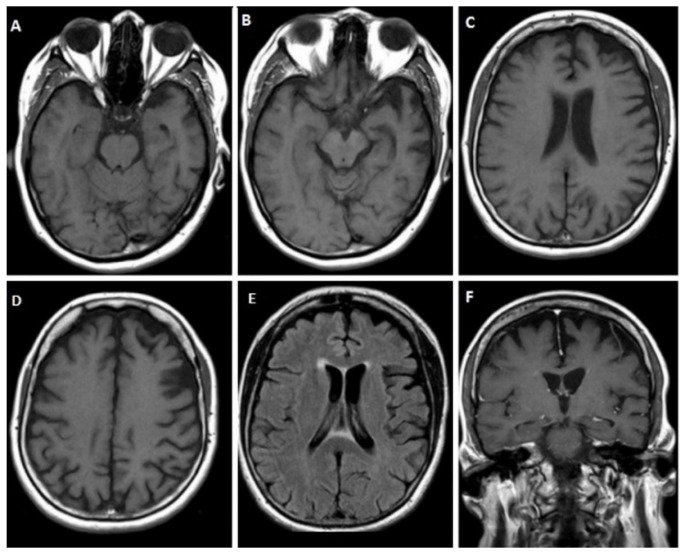
Brain MRI of case 1. T1 sequence (**A**–**D**) and fluid attenuated inversion recovery (FLAIR) (**E**) showing cortical atrophy in the frontal and temporal lobes, with left hemisphere being more affected, as well as coronal T1 section (**F**) showing relative preservation of the hippocampus.

A 61-year-old female patient was admitted to our department for progressive language impairment over the past 1–2 years. She had word-finding difficulty, with intact comprehension, an absence of phonological speech or grammar errors, no other significant cognitive/behavioral disturbances, and preserved activities of daily living. Neuropsychological assessment revealed mild cognitive impairment, with an MMSE score of 25/30 and a FAB score of 12/18. Her writing skills and phrase and sentence repetition were preserved, too. On magnetic resonance imaging (MRI), moderate cortical atrophy in the frontal and temporal lobes, with left predominance and relative preservation of the hippocampus, was apparent (Figure 1). CSF biomarker analysis revealed increased τ_T_ = 511.6 pg/mL, normal τ_P-181_ = 28.7 pg/mL, decreased Aβ_42_ = 504.2 pg/mL, but normal Aβ_42_/Aβ_40_ = 0.1 and decreased τ_P-181_/τ_T_ = 0.056 ratio. TDP-43 = 4.46 pg/mL and the formula TDP-43 × τ_Τ_/τ_P-181_ = 79.5 were increased. Genetic testing revealed a c.463-2A > G splice site variant—heterozygous mutation in the *GRN* gene. The patient also had more than a 2-fold decrease in plasma progranulin levels, as described elsewhere [28].

#### 3.1.2. Case 2

**Figure 2:**
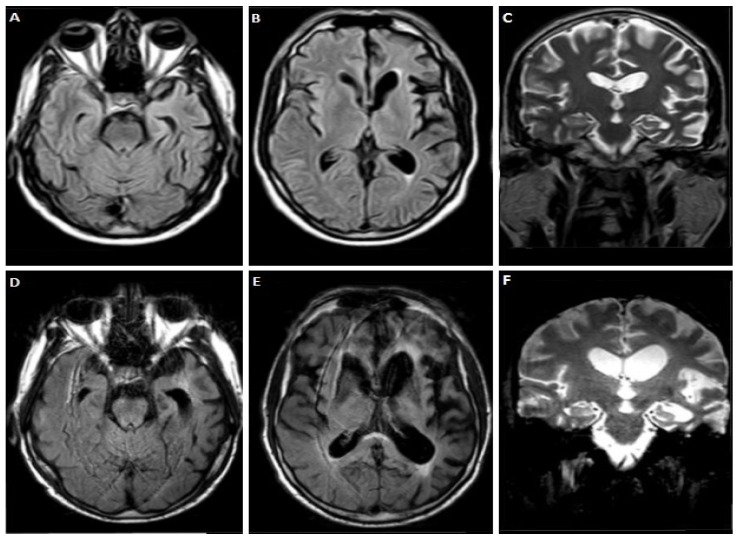
Brain MRI of case 2 at age 57 (**A**–**C**) and 60 (**D**–**F**) showing some degree of cortical atrophy in frontal and temporal lobes was observed with left predominance that deteriorated significantly three years later.

A 60-year-old male patient, with a family history of dementia on his mother and maternal grandmother’s side, presented with language impairment over the last 10 years. Initially, he had difficulty naming objects and writing. Three years later he developed effortful speech with sound errors, decreased fluency, and memory complaints and disorientation. At the age of 55, a dysexecutive syndrome as well as behavioral disturbances (overeating and irritability) appeared. Gradually, he became almost mute, while bradykinesia and tremor on the right hand appeared. At the time of his admission, he was completely mute, and unable to eat. Neurological examination revealed diffuse muscle atrophies without fasciculations, bilateral Parkinsonian syndrome (more severe in the right limbs), pyramidal syndrome with spasticity, hyperreflexia, and Babinski sign bilaterally. Neuropsychological assessment was impossible to perform. MRI performed at the age of 57 revealed significant cortical atrophy in the frontal and temporal lobes with left predominance (Figure 2A–C), with further deterioration three years later, as shown in (Figure 2D–F). CSF biomarker analysis revealed increased τ_T_ = 565.12 pg/mL, normal τ_P-181_ = 41.9 pg/mL, Aβ_42_ = 640 pg/m and Aβ_42_/Aβ_40_ = 0.11, while τ_P-181_/τ_T_ = 0.074, was decreased. TDP-43 = 5.92 pg/mL and formula TDP-43 × τ_Τ_/τ_P-181_ = 79.9 were increased. Genetic testing revealed a c.934-1G > A splice site variant—heterozygous mutation on the *GRN* gene, and similarly, a decrease in plasma progranulin levels, as in case 1.

#### 3.1.3. Case 3

**Figure 3:**
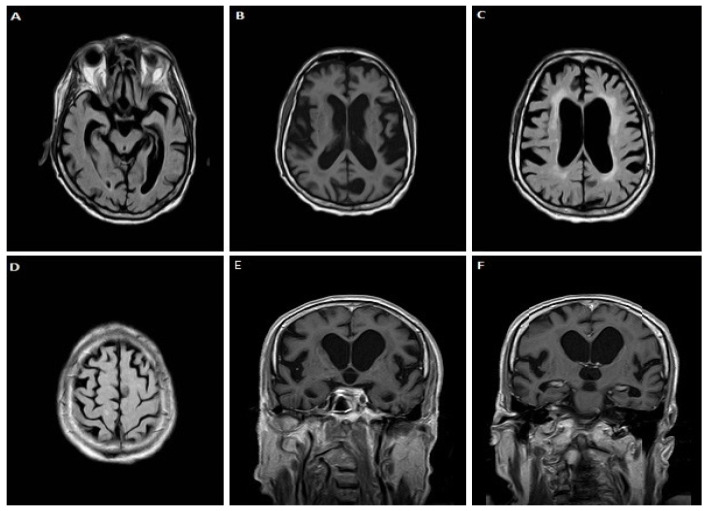
Brain MRI of case 3. FLAIR images (**A**,**C**,**D**) show severe cortical atrophy in frontal and temporal lobes. In axial (**B**) and coronal T1 section (**E**,**F**) atrophy of the perisylvian and parietal areas is also evident.

A 72-year-old male patient, with a family history of motor neuron disease (brother), gradually developed language (phonological speech errors, impaired comprehension, inability to write, severe difficulty in naming objects and reduced phonemic and categorical fluency) and memory impairment, bradykinesia and stereotypical movements of the right hand over the last two years. Three months before admission, his symptoms aggravated. Neurological examination revealed bilateral akinetic-rigid Parkinsonian syndrome, mild pyramidal signs (mainly on the right lower limb with hyperreflexia and extensor plantar response) and apraxia. Neuropsychological assessment revealed severe diffuse cognitive deficits (MMSE score 5/30, FAB score 1/18). On MRI, severe cortical atrophy in frontal and temporal lobes was observed (Figure 3). CSF biomarker analysis revealed normal τ_T_ = 316.16 pg/mL and τ_P-181_ = 27.2 pg/mL, decreased Aβ_42_ = 501.51 pg/mL but normal Aβ_42_/Aβ_40_ = 0.14 and low τ_P-181_/τ_T_ = 0.086 ratio. TDP-43 levels were 5.74 pg/mL and TDP-43 × τ_Τ_/τ_P-181_ = 66.7, both increased. The genetic testing revealed a pathologic hexanucleotide repeat expansion in the *C9orf72* gene.

#### 3.1.4. Case 4

**Figure 4:**
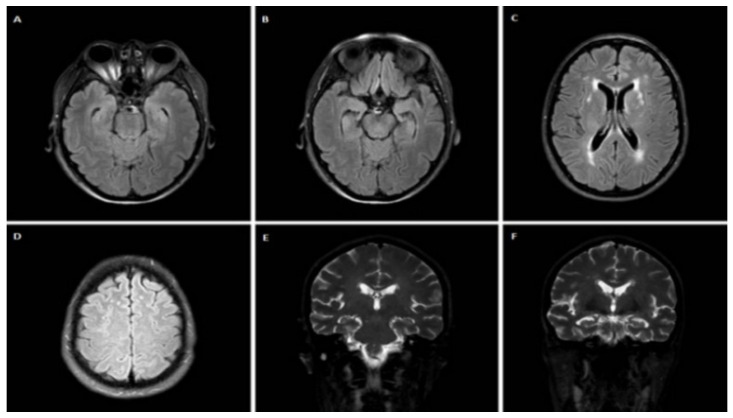
Brain MRI of case 4. Axial FLAIR images (**A**–**D**) and coronal T2 images (**E**,**F**) reveal hyperintense lesions attributed to chronic small vessel ischemia (Fazekas II) and some degree of cortical atrophy in temporal lobes.

A 54-year-old female patient, with a medical history of depression with psychotic features was referred for evaluation. She had a family history of dementia (mother) and symptoms of motor neuron disease (two maternal uncles). The patient presented with apathy, inertia, disinhibition, loss of empathy and sympathy, unusual stereotyped and perseverative behaviors, and hyperorality with overeating of carbohydrates during the last two and a half years. One and a half year before her admission, she also developed inappropriate crying and a language impairment. Neurological examination revealed severely impaired non-fluent speech with single-word answers, severe disorientation in time and space, and frontal gait disorder. Neuropsychological assessment revealed moderate cognitive deficits with an MMSE score of 15/30 and a FAB score of 6/18. On MRI, some T2 hyperintense lesions attributed to chronic small vessel ischemia (Fazekas II), and mild cortical atrophy of the temporal lobes was observed (Figure 4). CSF biomarker analysis revealed normal τ_T_ = 250.3 pg/mL, τ_P-181_ = 35.45 pg/mL and Aβ_42_ = 935.95 pg/mL, while τ_P-181_/τ_T_ ratio was 0.14. The levels of TDP-43 = 2.48 pg/mL and TDP-43 × τ_Τ_/τ_P-181_ = 17.5 were also normal. Genetic testing, however, revealed a hexanucleotide repeat expansion in the *C9orf72* gene.

#### 3.1.5. Case 5

**Figure 5:**
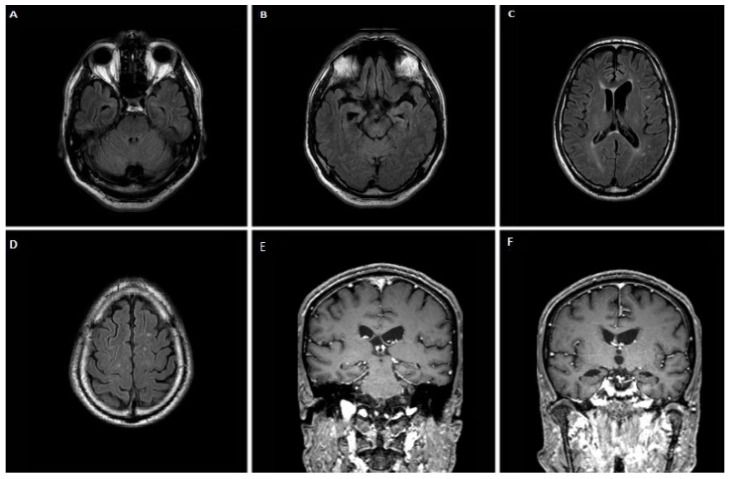
Brain MRI of case 5. Axial FLAIR images (**A**–**D**) and coronal T1 images (**E**,**F**) reveal mild frontal lobe atrophy.

A 62-year-old male patient, with a medical history of arterial hypertension and an operation for a herniated lumbar disc at the L4-L5 level, was referred for evaluation of neuropsychiatric symptoms. He had a family history of “presenile dementia” in his mother, ALS of his older brother, who died at the age of 53, and behavioral symptoms of another brother. The patient presented with progressively deteriorating steppage gait 20 years ago. A year before admission, the patient was unable to walk independently. Additionally, he developed obsessive behavior, aggression, palilalia and apathy. His neuropsychological evaluation revealed severe cognitive impairment, suggestive of predominant frontal lobe dysfunction, MMSE: 2 and FAB: 0 (not applicable). Brain MRI showed mild frontal lobe atrophy (Figure 5). EMG showed diffuse myopathic findings and mild spontaneous activity. Due to the non-AD biomarker profile, the family history of dementia and ALS in members of consecutive generations, which is indicative of a possible autosomal dominant inheritance pattern and the clinical and laboratory findings suggesting myopathy, a high suspicion of a possible *VCP*-associated syndrome was raised. Genetic testing (DNA isolated from skeletal muscle) revealed a pathogenic heterozygous missense mutation p.R159H (c.476G > A) in the *VCP* gene, confirming the diagnosis of IBMPFD [29].

Lumbar puncture and CSF biomarker analysie were performed one year later and revealed normal τ_T_ = 356.75 pg/mL, τ_P-181_ = 32.77 pg/mL, low Aβ_42_ = 431 pg/mL, but normal Aβ_42_/Aβ_40_ = 0.1 and reduced ratio τ_P-181_/τ_T_ = 0.091, while TDP-43 levels = 5.27 pg/mL, and TDP-43 × τ_Τ_/τ_P-181_ = 57.3 were increased.

#### 3.1.6. Case 6

**Figure 6:**
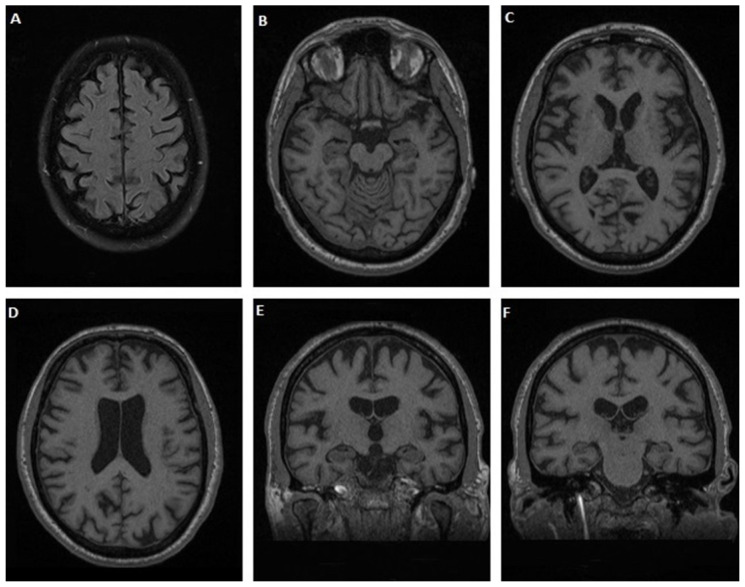
Brain MRI of case 6. Axial (**A**–**D**) and coronal (**E**,**F**) T1 images showing significant cortical atrophy in frontal, parietal and temporal lobes.

A 41-year-old male patient, with a medical history of dyslipidemia was admitted to the neurology department for delusions of religious content over the past 12 years. Moreover, apathy, a change in eating habits with carbohydrate craving and weight gain gradually developed, while dysarthria, executive deficits, stereotypical behaviors and inappropriate laughter appeared over the last two years. Finally, difficulty in swallowing liquids was noticed six months before admission. Neurological examination revealed pseudobulbar dysarthria, an absence of pharyngeal reflex, bilateral pyramidal syndrome and fasciculations in tongue and back muscles. Neuropsychological assessment revealed mild to moderate frontal cognitive deficits with an MMSE score 28/30, and a FAB score 15/18. Neurophysiological examination could not confirm lower motor neuron involvement. On MRI, significant cortical atrophy in frontal, parietal and temporal lobes was observed (Figure 6). CSF biomarker analysis revealed normal τ_T_ = 328.05 pg/mL, τ_P-181_ = 32.07 pg/mL, decreased Aβ_42_ = 397.1 pg/mL, with normal ratio Aβ_42_/Aβ_40_ = 0.17 and decreased τ_P-181_/τ_T_ = 0.098, while TDP-43 levels 5.74 pg/mL and the formula TDP-43 × τ_Τ_/τ_P-181_ = 57.66 were increased. Genetic testing finally revealed a hexanucleotide repeat expansion in the *C9orf72* gene.

Table 2 summarizes the CSF biomarker profile for all cases. All cases had normal τ_P-181_ and only two out of six cases had increased τ_T_. Four out of six cases had decreased Aβ_42_; however, all had a normal Aβ_42_/Aβ_40_ ratio. Five out of six patients had increased TDP-43 and formula (TDP-43 × τ_Τ_/τ_P-181_) values, while τ_P-181_/τ_T_ was abnormal in all.

## 4. Discussion

Herein, we present six patients with clinical, neuropsychological and imaging data fulfilling the criteria of Rascovsky et al. [24] and Gorno-Tempini et al. [25] for probable FTD, in which, due to the genetic findings, patients were classified as definite FTLD with TDP-43 histopathology. Three of our cases had a pathogenic hexanucleotide repeat expansion in the *C9orf72* gene; two had a novel mutation in the *GRN* gene which led to more than a 2-fold decrease of plasma progranulin levels, as expected [28,30]; meanwhile, one patient harbored a pathogenic mutation in the *VCP* gene. Although small in number, this group of patients is indicative of the most common mutations found in the Greek population [28,31].

In this group of well-characterized genetic FTLD patients, established CSF biomarkers for AD diagnosis (τ_T_, τ_P-181_, Aβ_42_ and Aβ_40_) and a novel emerging (TDP-43) biomarker were analyzed. Combinations of these biomarkers as the formula TDP-43 × τ_Τ_/τ_P-181_ and the ratio τ_P-181_/τ_Τ_ were also calculated, as previously suggested [18,22].

According to the AT(N) system proposed by the National Institute of Aging and Alzheimer’s Association (NIA–AA) Research Framework group, AD biomarkers are divided into those with molecular specificity for AD, namely τ_P-181_, Aβ_42_ and Aβ_42_/Aβ_40_ ratio, and those with neurodegeneration, τ_T_ in the present case [15]. All presented cases had a biomarker profile of either A-T-(N+) or A-T-(N−) according to CSF data, compatible with a “non-Alzheimer’s pathological change” profile. Only one patient (case 4) had a “normal” biomarker profile.

In four out of the six presented cases, Aβ_42_ was reduced, but the use of Aβ_42_/Aβ_40_ ratio restored normality, ruling out amyloidopathy (A). The Aβ_42_/Aβ_40_ ratio has been suggested to better reflect the presence or absence of amyloid pathology than Aβ_42_ alone. Furthermore, the Aβ_42_/Aβ_40_ ratio correlates better with amyloid load in PET (positron emission tomography) [32], as it has been found to be reduced in some cases of FTD and vascular dementia (VD), but most commonly in dementia with Lewy bodies (DLB) [33,34,35,36].

The main findings of our study, however, were the increase of CSF TDP-43 levels (higher than the above cut-off value) in five out of six cases, and the increased values of the TDP-43 × τ_Τ_/τ_P-181_ formula. TDP-43 is a protein, which may be suggestive and specific of TDP-43 proteinopathy. Additionally, the τ_P-181_/τ_T_ ratio was decreased, which in non-AD (FTD) patients could be considered as an indirect indication for a non-tauopathic pathology [22,23].

TDP-43 aggregation appears in approximately 50% of all FTLD cases (familial and sporadic) and in 97% of all ALS cases [37]. Mutations associated with TDP-43 histopathology have been documented for *C9orf72*, *GRN*, *VCP*, and *TARDBP* genes for FTLD and the FTD-ALS spectrum [38]. TDP-43 was initially considered as an intracellular/intranuclear protein; however, it is now recognized as an important protein for the existence and wellbeing of cells, through its various functions in RNA metabolism and homeostasis. Apart from FTLD, abnormalities in this protein’s homeostasis are associated with other severe neurodegenerative disorders [39]. Abnormal TDP-43 seems to cause neurotoxicity by various pathogenic processes, although the exact pathophysiological mechanisms that eventually lead to neurodegeneration remain unclear [1].

Although thought to exist as an intracellular/intranuclear protein, recent studies using ELISA and Western blot techniques suggest that TDP-43 can also be detected in extracellular fluids such as plasma and CSF [40].

The results of the present study indicate that CSF TDP-43 levels were increased in five out of six patients with TDP-43 histopathology-associated mutations. Case 4, with a *C9orf72* pathological repeat expansion, represents an exception. Nonetheless, the proposed mechanisms through which *C9orf72* mutations cause neurodegenerative changes are three: *C9orf72* loss-of-function through haploinsufficiency, RNA toxic gain of function, and gain of function through the aggregation of toxic dipeptide repeat proteins [41]. The first two mechanisms cause neurodegeneration related to TDP-43 aggregation. The “gain of function” hypothesis due to the formation and accumulation of dipeptide repeats (DPRs) causes neurodegeneration via repeat-associated non-AUG (RAN) translation of the hexanucleotide GGGGCC from both sense and antisense strands [42]. As a result, there is a production of five distinct DPRs from sense {(poly-GA (glycine–alanine), poly-GP (glycine–proline), poly-GR (glycine–arginine) and antisense poly-GP (glycine–proline), as well as from poly-PR (proline–arginine) and poly-PA (proline–alanine)} strands [43,44]. These DPRs are amyloidogenic, and accumulate in different parts of the central nervous system without TDP-43 aggregation [43,44,45]. The pathogenic contribution of DPR-associated toxicity to disease progression remains unknown. As there is no aggregated TDP-43 in neuronal cytoplasmic inclusions in such patients, we can assume that the TDP-43 concentration in their CSF would be normal. This reasoning provides an explanation for the normal values of TDP-43 and formula in case 4.

TDP-43 abnormalities have also been observed in other neurodegenerative diseases as well, mainly in ALS, the other end of the ALS-FTD spectrum, but also in AD, limbic-predominant age-related TDP-43 encephalopathy (LATE), cerebral age-related TDP-43 with sclerosis (CARTS), and some other even rarer conditions [27,46,47]. Reports on CSF TDP-43 levels are mostly derived from patient cohorts of sporadic FTD cases, with inadequate (74%) clinicopathological concordance rates [48], resulting in relatively low sensitivities and specificities vs. controls. Thus, we enrolled genetic cases in order to reach safer conclusions. Sensitivities and specificities compared with other dementing disorders would have to be investigated as well.

There are also other biomarkers that can be used to differentiate FTD patients from patients with other neurodegenerative diseases, such as NfL [49]. However, NfL, total tau protein and the specific patterns of atrophy, are considered biomarkers of neurodegeneration in general [15]. The aim of the current study was to assess the diagnostic value of CSF TDP-43 protein as a biomarker with molecular specificity for FTD. Therefore, we combined TDP-43 with established AD biomarkers only, mainly to exclude AD, which often enters into a FTD differential diagnosis.

The current study has certain limitations. TDP-43 pathology can only be presumed from genetic mutations, since it is an ante-mortem study. Another disadvantage is the small number of patients, coming from a single-center study that included only patients of Greek origin; however, the mutations that the patients harbored are indicative of the most commonly observed in TDP-43-related FTLD found in the Greek population [28,31].

Our study also has some important advantages. We describe patients that have undergone extended clinical, neuropsychological and imaging examinations, fulfilling the criteria of Rascovsky et al. [24] and Gorno-Tempini et al. [25] for probable FTD, while they are classified as definite FTLD with TDP-43 underlying pathology, due to the genetic findings. This homogeneous genetic FTD cohort may serve as a very good example of how relatively safe conclusions may be reached through personalized and precision medicine. Further studies with larger patient cohorts are nevertheless required to corroborate our results.

## 5. Conclusions

Based on the results of the present personalized case series study, which is nonetheless indicative of the mutational profile of the population of Greece, the following conclusions can be drawn:

- Established AD biomarkers are reliable indicators for the exclusion of AD pathology in genetic FTLD.
- A decreased τ_P-181_/τ_Τ_ ratio may be useful as an indirect marker for non-tauopathic pathology.
- TDP-43 alone and combined with tau proteins, could be a useful tool for the diagnosis of genetic FTD patients with TDP-43 underlying pathology.

Future studies with larger and well characterized patient cohorts are needed to further explore the diagnostic potential of these biomarkers in differentiating TDP-43 proteinopathy from non-TDP-43-related pathologies.

## Figures and Tables

**Table 1:** Cut-off values of CSF biomarkers applied at our laboratory [18,23].

CSF Biomarker	Cut-off Values *
total tau protein (τ_T_)	<376 pg/mL
tau phosphorylated at threonine-181 (τ_P-181_)	<57 pg/mL
amyloid-β peptide with 42 amino acids (Aβ_42_)	>580 pg/mL
Aβ_42_/Aβ_40_	>0.067
τ_P-181_/τ_T_	>0.165
TDP-43	<3.73 pg/mL
TDP-43 × τ_Τ_/τ_P-181_	<20.5

* From Paraskevas et al., 2017; Bourbouli et al., 2017.

**Table 2:** CSF biomarker levels in the presented cases.

Case	τ_T_ (pg/mL)	τ_P-181_ (pg/mL)	Aβ_42_ (pg/mL)	Aβ_42_/Aβ_40_	τ_P-181_/τ_T_	TDP-43 (pg/mL)	Formula (f)
1	**511.6**	28.7	**504.2**	0.10	**0.056**	**4.46**	**79.5**
2	**565.1**	41.9	640	0.11	**0.074**	**5.92**	**79.9**
3	316.2	27.2	**501.5**	0.14	**0.086**	**5.74**	**66.7**
4	250.3	35.5	935.9	-	**0.140**	2.48	17.5
5	356.8	32.8	**431**	0.10	**0.091**	**5.27**	**57.3**
6	328.1	32.1	**397.1**	0.17	**0.098**	**5.74**	**57.7**

τ_T_: total tau protein; τ_P-181_: tau phosphorylated at threonine-181; Aβ_42_: amyloid-β peptide with 42 amino acids; Aβ_40_: amyloid-β peptide with 40 amino acids; f: TDP-43 × τ_Τ_/τ_P-181_; pathological values are highlighted in bold.

## Data Availability

The data presented in this study are available upon reasonable request from the corresponding author. The data are not publicly available due to privacy restrictions.
